# ^1^H HR-MAS NMR Spectroscopy and the Metabolite Determination of Typical Foods in Mediterranean Diet

**DOI:** 10.1155/2015/175696

**Published:** 2015-10-01

**Authors:** Carmelo Corsaro, Domenico Mallamace, Sebastiano Vasi, Vincenzo Ferrantelli, Giacomo Dugo, Nicola Cicero

**Affiliations:** ^1^Istituto per i Processi Chimico-Fisici del CNR di Messina, Viale F. Stagno D'Alcontres 37, 98158 Messina, Italy; ^2^Dipartimento di Fisica e Scienze della Terra, Università di Messina, Viale F. Stagno D'Alcontres 31, 98166 Messina, Italy; ^3^Dipartimento di Scienze dell'Ambiente, della Sicurezza, del Territorio, degli Alimenti e della Salute, Università di Messina, Viale F. Stagno d'Alcontres 31, 98166 Messina, Italy; ^4^Istituto Zooprofilattico Sperimentale della Sicilia “A. Mirri”, Via G. Marinuzzi 3, 90129 Palermo, Italy; ^5^Science4life SRL Academic Spin-off, Università di Messina, Viale F. Stagno D'Alcontres 31, 98166 Messina, Italy

## Abstract

NMR spectroscopy has become an experimental technique widely used in food science. The experimental procedures that allow precise and quantitative analysis on different foods are relatively simple. For a better sensitivity and resolution, NMR spectroscopy is usually applied to liquid sample by means of extraction procedures that can be addressed to the observation of particular compounds. For the study of semisolid systems such as intact tissues, High-Resolution Magic Angle Spinning (HR-MAS) has received great attention within the biomedical area and beyond. Metabolic profiling and metabolism changes can be investigated both in animal organs and in foods. In this work we present a proton HR-MAS NMR study on the typical vegetable foods of Mediterranean diet such as the Protected Geographical Indication (PGI) cherry tomato of Pachino, the PGI Interdonato lemon of Messina, several Protected Designation of Origin (PDO) extra virgin olive oils from Sicily, and the Traditional Italian Food Product (PAT) red garlic of Nubia. We were able to identify and quantify the main metabolites within the studied systems that can be used for their characterization and authentication.

## 1. Introduction

In the last two decades, there was a remarkable increase in studies concerning food science performed by means of Nuclear Magnetic Resonance (NMR) spectroscopy [1, 2]. The reason for NMR success in food analysis lies essentially in the possibility to study complex matrices, obtaining a large number of information on metabolites within a single experiment, with minimal or no sample preparation [2]. In fact, even if other widely used analytical techniques such as Gas Chromatography (GC) have a higher sensitivity, they need quite sophisticated extraction procedures [3, 4]. Furthermore, advanced NMR hardware and user-friendly software have been developed as well as bidimensional techniques that allow easy metabolite identification [5]. The area below each proton NMR signal is directly proportional to the numbers of nuclei so its knowledge allows the determination of the quantitative chemical composition. Finally, its use in synergy with multivariate statistical analyses permitted a number of relevant studies on food metabolomics and chemometrics especially after the increasing needs for the control on food quality and safety [6, 7].

In order to obtain good quality NMR spectra, the system under study must be liquid. In fact, nonliquid systems are characterized by strong anisotropic interactions that cannot be averaged out and produce unresolved broad peaks. In particular, the NMR spectral sensitivity and resolution are limited by those mechanisms that provoke line-broadening effects also with high magnetic fields. Dipolar coupling and susceptibility heterogeneity are examples of these kinds of mechanisms that occur within biological samples. Therefore, one technique that can average multiple line-broadening mechanisms was developed [8] to limit and resolve these problems, particularly strong for solid-state NMR. This technique is known as magic angle spinning (MAS) NMR spectroscopy (see Section 2) and, taking advantage of “geometric constrains,” can be used to acquire high-resolution (HR) spectra of heterogeneous samples such as tissues and cells. This allows the determination of the metabolic profile of the studied system under the considered conditions. Therefore, HR-MAS NMR has become more popular in food science and in the biological and biomedical fields [9, 10].

As a matter of fact, many studies performed by means of HR-MAS on different organs and tissues have been reported, demonstrating—for example, the ability of this technique to discriminate between malignant and benign disease [11–13]. Moreover, the possibility to follow the metabolic changes has led to apply the HR-MAS technique in different fields ranging from the characterization and authentication of different foods [14] to the study of the cellulose degradation happening over centuries in ancient documents [15].

The experimental results we present in this work concern the application of this interesting and powerful technique to the study of the metabolic profile of some typical foods of the Mediterranean diet. In the early 1960s, in Greece and southern Italy, adult life expectancy was among the highest in the world and rates of coronary heart disease, certain cancers, and other diet-related chronic diseases were among the lowest. This was attributed to the particular diet adopted in those regions and today known as Mediterranean diet [17].

The Mediterranean diet is principally characterized by the consumption of olive oil and wine together with numerous plant foods (vegetables, breads, other forms of cereals, potatoes, beans, nuts, and seeds), fresh fruit (e.g., citrus), fish, and cheese. Poultry is consumed from low to moderate amounts; zero to four eggs are consumed weekly and red meat is consumed in low amounts. Furthermore, garlic, onions, and herbs were used as condiments. This diet is able to provide all of the known essential micronutrients (i.e., vitamins and minerals), fiber, and other plant food substances believed to promote health [17]. It is noteworthy that the Mediterranean diet was inscribed in 2013 on the Representative List of the Intangible Cultural Heritage of Humanity (UNESCO).

Among the different foods of the Mediterranean diet, we focused our attention on four important food products characterized by a Protected Geographical Status [18]: the PGI (Protected Geographical Indication) cherry tomato of Pachino, the PGI Interdonato lemon of Messina, several PDO (Protected Designation of Origin) extra virgin olive oils (eVOOs) from Sicily, and the PAT (Traditional Italian Food Product) red garlic of Nubia.

The European Union (EU) has restrictive laws about the food safety policy aimed at protecting consumer health and interests while guaranteeing the smooth operation of the single market. In particular, the EU ensures that control standards are established and adhered to regarding food and food product hygiene, animal health and welfare, and plant health and preventing the risk of contamination from external substances. It also establishes the bases for an appropriate labelling, in line with the approach “From the Farm to the Fork,” thereby guaranteeing a high level of safety for foodstuffs and food products marketed within the EU, at all stages of the production and distribution chains.

Indeed, in this paper we present HR-MAS NMR results on the mentioned four typical food products of the Mediterranean diet. The increasing demand of quality control by consumers pushes the development analytical techniques able to characterize the metabolic profile of a particular food. We were able to identify and quantify the main metabolites within the studied systems that can be considered their fingerprint. In fact, the used technique can reveal and quantify a number of metabolites even on few amounts of samples and without any chemical treatment. In spite of its quite low sensitivity, the rapidity and easiness of the HR-MAS technique, together with the reduction of chemical consumption and waste production, make the methodology very attractive for industry.

## 2. Materials and Methods

### 2.1. Instrumental

^1^H one and two-dimensional NMR experiments were conducted at atmospheric pressure by using a Bruker Avance spectrometer operating at 700 MHz, ^1^H resonance frequency, in the experimental configuration known as magic angle spinning (MAS). This technique was developed to reduce the two main line-broadening mechanisms that are important in acquiring spectra of a tissue or cell sample, namely, dipolar coupling and heterogeneous isotropic susceptibility [8]. Spinning the sample at the magic angle *θ* ~ 54.74° by few thousands of Hertz averages, these interactions to zero and high-resolution spectra can be achieved for semisolids samples.

Our experiments were performed at the temperature of 300 K calibrated against the standard CH_3_OH reference (4% CH_3_OH in CD_3_OD) with an accuracy of 0.2 K. Temperature calibration is very important for this kind of experiments because of the heat produced by the high rotational speed. In fact, the real sample temperature is higher with respect to that read by the thermocouple. For each experiment we use a 4 mm-diameter zirconia sample holder (rotor) with a spherical insert for a total volume of 50 *μ*L and a Kel-F rotor cap. We use deuterated solvents (D_2_O and CDCl_3_) in order to have a lock signal for a chemical shift reference and for a fine optimization of the static magnetic field homogeneity. Furthermore, the use of deuterated solvent is necessary in order to avoid any excessive proton signal from the solvent itself. In aqueous preparation we use a 1 mM solution of D_2_O with 2,2-dimethyl-2-silapentane-5-sulfonate (DSS) as an internal standard for the quantification of the assigned metabolites. High purity reagents were bought from Sigma-Aldrich Co. (Saint Luis, MO, USA). The acquired spectra were processed (Fourier transform, phase correction, and baseline adjustment) by means of the standard routines of the software package Xwinnmr version 3.5 (Bruker Biospin, Deutschland). Peaks assignment was performed by means of literature data and of a well-established software package: NMR Suite Professional version 7.1 (Chenomx, Alberta, Canada). This latter software is based on a highly sophisticated targeted profiling technology which allows an easy deconvolution of complex NMR spectra and the corresponding quantification of the identified compounds. For the complete and unambiguous assignment of some compounds we performed standard two-dimensional NMR techniques such as COSY and HSQC. For those metabolites not included in the software database (such as gallic acid) (see Section 3.2) and also for a confirmation of the Chenomx output, we used the standard Bruker program “nmrquant” for the quantification of metabolites. The NMR technique is less sensitive with respect to other well-established analytical techniques such as GC and ICP-MS (Inductively Coupled Plasma Mass Spectrometry) [19] and can quantify metabolites whose concentration is usually above one part per million. The quantification is obtained by using a reference compound of known concentration within the studied solution (1 mM DSS in D_2_O in our case). The area below each proton signal is proportional to the amount of the corresponding substance, so that by the knowledge of the chemical structure and molecular weight of the assigned metabolites, it is possible to obtain the molar concentration by a simple proportion between the peak areas. By means of the Chenomx software, all the spectral contributions belonging to the considered molecule should fit the experimental spectrum whereas by using nmrquant, the most intense and resolved peaks should be used for metabolites quantification. Finally, we can state that the advantages of the used method rely on the rapidity of the analysis without the needs for any sample treatment that allows, at least in principle, the reuse of the sample. Furthermore, the method is precise and allows observing a great number of compounds simultaneously. On the contrary, the method is not very sensitive for the detection of compounds whose concentration is below one part per million. Precise extraction procedures are needed in order to observe particular compounds (or secondary metabolites) at very low concentration.

In the following subsections, we describe the sample preparation and experimental procedures for each single food products we have analyzed.

### 2.2. PGI Cherry Tomato of Pachino

The PGI cherry tomato of Pachino is produced within an area located in the south east of Sicily (Italy) that includes the entire municipality of Pachino and Portopalo di Capo Passero and part of the territories of Noto and Ispica. We analyzed 14 cherry tomato samples of Pachino and 14 of dubious provenience (non-Pachino) including 2 coming directly from Beijing (China) [20]. For a statistically significant outcome we analyzed at least 5 samples for each kind of tomato. The PGI cherry tomatoes of Pachino were provided by Istituto Zooprofilattico Sperimentale della Sicilia “A. Mirri” (http://www.izssicilia.it/) which is the official institution recognized by the EU as to able to certificate this food product.

All samples were studied in the red stage which is when more than 90% of the surface, in the aggregate, is red. In doing so we reduce the eventual metabolic differences due to different ripening stage [21, 22]. We diluted 6 mg of freeze-dried tomato in 100 *μ*L of a 1 mM solution of DSS in D_2_O. Then, we vortexed for a couple of minutes and put fifty microliters into the rotor that in this case was spun at 6000 Hz. The duration of the hard pulse was of 8 *μ*s with a relative attenuation of 3 dB, the spectral width was 10 kHz, the acquisition time was 2.9 s, the points in the time domain were 64 k, the number of transient was 128, and the relaxation time was 2 s for a total time of about 10 min per experiment. For the reduction of the residual signal of water we use the standard Bruker presaturation pulse sequence* zgpr* with a presaturation pulse attenuation of 60 dB. When processing the spectra we considered 32 k points in the frequency domain.

### 2.3. PGI Interdonato Lemon of Messina

The PGI Interdonato lemon of Messina is traditionally also known as “limone fino” (fine lemon) and “limone speciale” (special lemon). Interdonato lemons have an oval shape with a yellow peel. Interdonato lemons own a strong fragrance and an acidic taste. They are rich in sugars, vitamin C, and flavonoids, which are very important in human metabolism. We have analyzed 10 different Interdonato lemon samples cultivated in Sicily and 10 cultivated in Turkey at the same ripening stage. For each sample we have repeated the measurements on 6 different replicates in order to have a statistically significant outcome. The PGI Interdonato lemons were provided by 4 different companies belonging to the “Consorzio di tutela del limone Interdonato di Sicilia IGP”. We extracted the lemon juice by a simple mechanical procedure and diluted 20 *μ*L of juice in 30 *μ*L of 1 mM DSS dissolved in D_2_O directly into the rotor with a spherical insert and a Kel-F rotor cap. The sample was kept at ambient temperature (300 K) by a cold *N*_2_ flow and a heating element. We used the following experimental parameters: rotor spinning rate 6000 Hz, duration of the hard pulse 8 *μ*s, spectral width 10 kHz, acquisition time 2.9 s, 64 k points in the time domain, 128 transients, and 2 s of relaxation time. We use the standard Bruker presaturation pulse sequence “zgpr” to achieve a reduction of the residual water signal. The total time necessary for each experiment was of about 10 min and we used 32 k points in the frequency domain for processing the spectra.

### 2.4. PDO Extra Virgin Olive Oil of Sicily

16 samples of different eVOOs were selected from different geographical areas of Sicily and in particular 8 from the province of Trapani (TP), 5 from that of Messina (ME), and 3 from that of Agrigento (AG). Among these we consider the following PDO cultivars: Valle del Belice (TP), Val di Mazara (TP), Valli Trapanesi (TP), and Valdemone (ME). All samples, after being carefully kept away from light and possible temperature changes that would alter the nature of the oils, were analyzed by taking 30 *μ*L of CDCl_3_ and 20 *μ*L of sample, placed in the rotor. The spectral width used was of 20 ppm (~14 kHz), the repetition time was 5 s, and the number of transients was 128. The duration of the hard pulse was of  5 *μ*s with an attenuation of 3 dB. The rotation speed of the rotor was set to 7000 Hz and the total time was of about 15 min per experiment.

**Figure 1:**
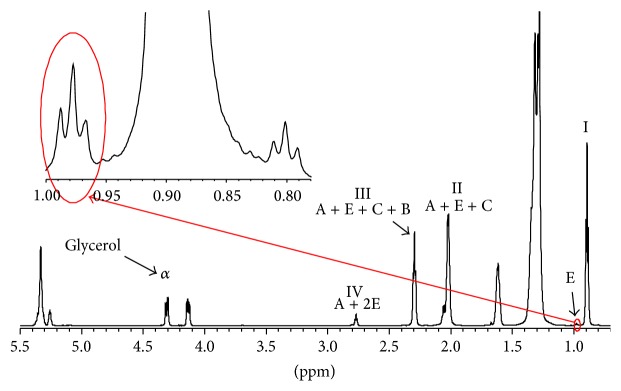
Typical proton NMR spectrum of eVOOs with highlighted the characteristic peaks of fatty acids and glycerol moieties used for the determination of fat percentage. In addition, the expansion shows the ^13^C satellites of the methyl peak at 0.85 ppm (I) superimposed to that of linolenic at 0.98 ppm (E).

The main signals of the typical proton spectrum of eVOOs come from fatty acids [23–25]. We used two different methods based on peaks integration for the determination of the fatty acids composition. In particular, Barison et al. [23] consider that all fatty acyl chains are esterified to a common moiety, glycerol, in order to form triacylglycerols. This means that it is possible to obtain the fatty acid composition by the inherent connection between the areas of the characteristic signals of each fatty acyl chain and one of the glycerol backbone (*α*) in the ^1^H NMR spectra (Figure 1, e.g., signals at 4.27 ppm) [23]. In particular, fatty acids can be esterified up to three times to the same glycerol moiety and this fact must be taken into account in order to calculate the correct amount. For example, it is possible to have up to three linolenic acid groups esterified to the same glycerol moiety so a ratio of 22.2 *α* glycerol to 100 linolenic acid hydrogens should be used when integrating the signal E (0.98 ppm) in Figure 1. Special attention should be given to the integral determination (limits, slope, etc.). For example, in our case (700 MHz) the ^13^C satellites of most intense peaks have to be subtracted by the integrated region in order to obtain correct values (e.g., as shown in the expansion of the linolenic (E) signal at 0.98 ppm reported in Figure 1) [24]. Accordingly, the percentage of linoleic (A) acid can be determined by integrating the signal at about 2.74 ppm (signal IV = A + 2E in Figure 1), which refers to the methylene hydrogens between two double bonds or olefins [23].

Therefore, by setting the integral of the chosen glycerol signal to 33.3, the relative area found for the signal at 2.74 ppm directly gives the percentage of linoleic plus linolenic acids. Since linoleic (A) and linolenic (E) acids have, respectively, two and four methylene hydrogens between olefins, and the percentage of linoleic acid is obtained by subtracting twice the content of linolenic acid that was previously determined [23]. The signal (II in Figure 1) at about 2.02 ppm refers to the methylene *α* olefin hydrogens of all unsaturated fatty acids, and being the ratio of 2 *α* glycerol hydrogens to 12 possible *α* olefin hydrogens, if the signal of *α* glycerol hydrogens is set to 16.7, the area of the signal at 2.02 ppm provides the percentage of all unsaturated fatty acids: linolenic (E), linoleic (A), and oleic (C). The percentage of oleic acid can be then obtained by subtracting from the value found, the contributions of the unsaturated acids previously obtained [23]. Finally, by setting again the integral of the chosen glycerol signal to 33.3, that of the signal at 2.28 ppm (from six *α* carbonyl hydrogens of all fatty acids esterified to the glycerol moiety) is approximately 100. Therefore, the percentage of saturated fatty acids can then be determined by subtracting from the area of the signal at 2.28 ppm, the contributions of the unsaturated oleic, linoleic, and linolenic acids found earlier.

The second method that we used was introduced by Vigli et al. [25] by considering that the content of linoleic acid can be determined by referring the intensity of its characteristic methyl signal at 0.95 ppm (signal E in Figure 1) to the intensity of the methyl signal at 0.85 ppm (signal labeled I in Figure 1) belonging to all acids except linolenic [25]. Consider

(1) $$\begin{array}{c} \left[\;\text{linolenic}\;\right]=\frac{E}{\left(E+I\right)}. \end{array}$$

The relative amount of linoleic acid can be determined by subtracting from the signal of diallylic protons at 2.73 ppm (signal IV in Figure 1) the relative amount of linolenic calculated earlier as well as the oleic acid content that can be determined by referring the allylic protons centered at 2.02 ppm (signal II in Figure 1) to all fatty chains as measured from the intensity of the C-2 protons around 2.3 ppm (signal III in Figure 1) [25]. One has

(2) linoleic=3IV−4E3IIIoleic=II2III−linoleic−linolenicsaturated=IE+I−linoleic−oleic−linolenic.

### 2.5. Red Garlic of Nubia

The red garlic of Nubia is cultivated within the municipality of Paceco (Trapani) and in particular in the Integral Natural Reserve of Saline in Trapani. It is a food product registered to the Italian Ministry of Agricultural, Food, and Forestry Policies as a Traditional Italian Food Product (PAT). We have prepared the garlic samples by accurately cutting a thin strip of about 20 mg, rolling it directly into the rotor together with 30 *μ*L of 1 mM DSS in D_2_O. In our one-dimensional experiments we used 32 k points in the time domain and a spectral width of 14 ppm (~10 kHz). The repetition time was set to 3 s and the number of transients was 256. The duration of the hard pulse was of 6.4 *μ*s with an attenuation of 3 dB. Also in this case, to reduce the residual water signal, we use the standard Bruker presaturation pulse sequence* zgpr*. The rotation speed of the rotor was set to 7000 Hz and the total duration was of about 20 min per experiment. We have performed the experiments on 6 samples and 6 different replicates in order to have a statistically significant outcome.

## 3. Results and Discussion

### 3.1. PGI Cherry Tomato of Pachino

Tomato (*Solanum lycopersicum*) is probably the most consumed fresh vegetable all over the world. Tomato is low in calories and shows antioxidant, antitumoral, and antidepressive properties [26] due to the relatively high concentration of lycopene, ascorbic acid, vitamin E, flavonoids, and so forth. The first tomato accredited by the PGI certificate (Council Regulation (EEC) number 2081/92) and one of the most counterfeit food product is the Sicilian cherry tomato of Pachino. Its special taste comes from the right combination of sugars, organic acids, free amino acids, and salts [20, 27].

**Figure 2:**
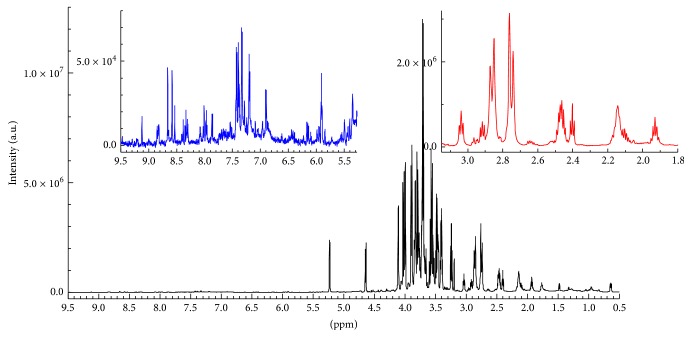
The complete typical proton spectrum of a PGI cherry tomato of Pachino sample is reported in the main plot. The expansions report the enlargement of the phenolic region (left side, blue line) and part of the aminoacidic region (right side, red line).

We were able to identify and quantify the molar concentration of the main metabolites that can be observable by means of our NMR technique. In such a way we aim to obtain a metabolic fingerprint of this protected foodstuff that allows for its characterization and authentication.  Figure 2 reports in the main plot the complete typical proton spectrum of a PGI cherry tomato of Pachino sample. The expansions are the enlargement of the phenolic region (left side, blue line) and part of the aminoacidic region (right side, red line). One can note the high resolution of the obtained spectra especially in the phenolic region that is usually characterized by high noise level.

We used for peaks assignment literature data [21, 22, 28] and the above mentioned software package NMR Suite Professional version 7.1 (Chenomx, AB, Canada) that allows also the determination of metabolites concentration [20]. We followed the same procedures also for the non-Pachino cherry tomato samples in order to execute a multivariate statistical analysis in terms of the Principal Components Analysis (PCA). This kind of multivariate statistical analysis, being based on an unsupervised pattern recognition technique, allows the identification of differences and similarities between NMR metabolic fingerprints.

In particular, NMR spectra were processed by means of a custom-written ProMetab 3.3 software [29] in MATLAB version R2009b (The Math Works, Natick, MA, USA); spectra were binned from 0.7 to 10.0 ppm with 0.005 ppm bin size; the residual water signal (4.65–4.95 ppm) was excluded; spectra were normalized to the total area and were generalized by log transformation (with a transformation parameter, *λ* = 10^−6^) to stabilize the variance across the spectral bins and to increase the weightings of the less intense peaks [29]. Finally we use the software package Unscrambler X version 10.0.1 (Camo Software AS, Oslo, NO) for the PCA analysis with cross validation; data were mean-centered and the singular value decomposition (SVD) algorithm was used.

**Figure 3:**
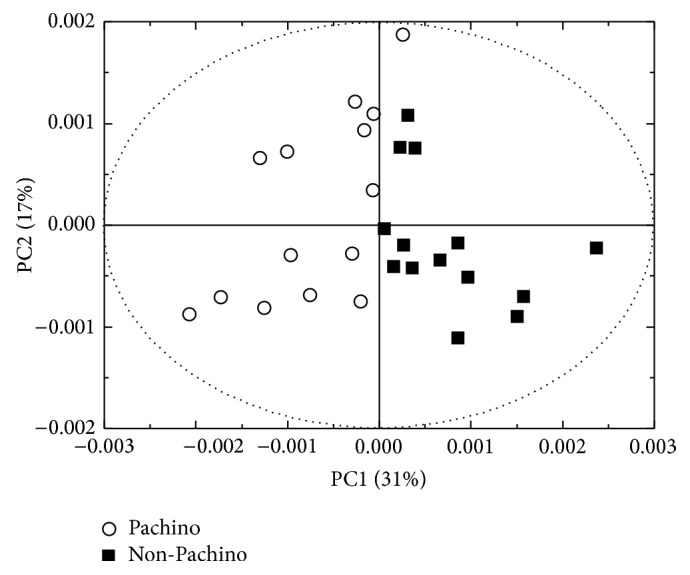
Score plot of the PCA analysis performed on cherry tomato samples. The Principal Component 1 (PC1) discriminates between Pachino and non-Pachino cherry tomatoes sample.

Our aim is to establish if there are any metabolites that can account for sample differentiation. We report in Figure 3 the results of the PCA analysis in the form of a score plot, where samples that are metabolically similar cluster together. As it can be noticed, the Principal Component 1 (PC1) is able to separate Pachino from non-Pachino cherry tomatoes except for one sample.

The final identification of the metabolites that can account for sample differentiation can indeed be obtained by analyzing the loadings corresponding to the PC1. The loadings of a Principal Component represent the weight by which each standardized original variable should be multiplied to get the component score. In particular, positive loadings values represent metabolites that are predominant in non-Pachino samples and vice versa [20]. Therefore, we were able to identify those metabolites whose concentration can determine the sample clustering. Student's *t-test* analysis, performed by means of the software package Microsoft Excel (Microsoft Co., WA, USA), allows to determine only the statistically significant (*p* value less than 0.05) changes of metabolites that we report in Table 1. Sugars, GABA, glutamic acid, trigonelline, tryptophan, and tyrosine concentration is higher in Pachino cherry tomatoes whereas that of alanine, guanosine, and methanol is higher in non-Pachino ones.

We want to stress that our result should be independent of factors such as the annual weather variations that can provoke some metabolic changes. In fact, it was shown that despite the marked variability showed only by antioxidants content, greenhouse-growing conditions in Sicily induce the accumulation of relatively high levels of ascorbic acid, phenolic compounds, and carotenoids in cherry tomatoes for most of the year [30]. Moreover, very recent studies on the response of tomato to constraining the intensity of solar radiation showed that the tomato plant's metabolism has a strong adaptation to cope with the limitation in light availability such as increasing the specific leaf area and reducing respiration. This was only of little concern to the fruit quality, because no effect of constraining the intensity of solar radiation on the concentration of total dry matter, sugars, and lycopene in the fruits was observed [31].

### 3.2. PGI Interdonato Lemon of Messina

Lemon (*Citrus limon* (L.) Burm.), similarly to tomato, is one of the most consumed fresh fruit. Lemon is low in calories and displays antioxidant and antineoplastic properties [32] that depend on the relatively high concentration of potassium, magnesium, calcium, vitamin C, phenolic compounds, and so forth. In particular, lemon is the third most important health-promoting fruit rich in phenolic compounds as well as vitamins, minerals, dietary fiber, essential oils, and carotenoids. Furthermore, it is widely used also by the food industry as raw materials or flavoring additives for a wide variety of products. Indeed, lemons have a strong commercial value for fresh products market and food industry [32].

The cultivar of our interest, known as “Interdonato,” represents a hybrid between a cedar and a lemon. It is cultivated in the province of Messina (Italy) within an area delimited by the Ionian Sea and the Peloritans Mountains and it is one of the few citrus accredited by the European PGI certificate (Commission Regulation (EC) number 1081/2009).

In particular, we have studied the one-dimensional proton spectrum of lemon juice for different samples of both PGI Interdonato lemon of Messina and Interdonato lemon from Turkey [33]. We have assigned and quantified the main metabolites that are present in these two hybrids by means of literature data [34] and the above mentioned Chenomx software package. In fact, other studies performed on the different tissues of lemon with the HR-MAS technique are present in literature [34] that agree with our results. However, in this mentioned study no metabolites quantification was performed.

**Figure 4:**
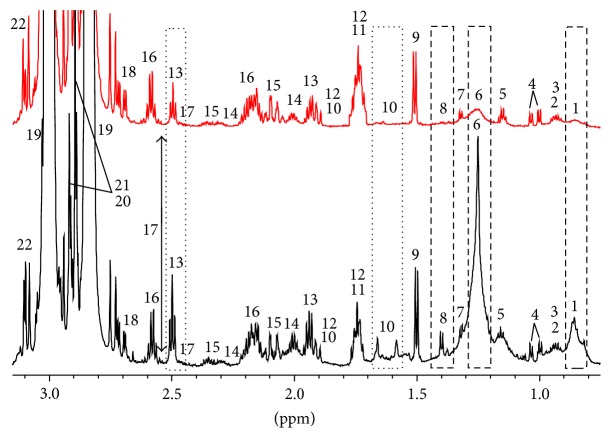
Comparison between the one-dimensional proton spectra of the lemon juice from PGI Interdonato lemon of Messina (red line) and Interdonato Turkish lemon (black line) in the region of amino acids. 1: saturated fatty acids, 2: isoleucine, 3: leucine, 4: valine, 5: ethanol, 6: unsaturated fatty acids, 7: threonine, 8: lactic acid, 9: alanine, 10: arginine, 11: DSS, 12: lysine, 13: GABA, 14: proline, 15: glutamic acid, 16: glutamine, 17: isocitric acid, 18: aspartic compound, 19: citric acid, 20: malic acid, 21: asparagine, and 22: stachydrine.

Figure 4 reports the comparison between the one-dimensional proton spectrum of the PGI Interdonato lemon (red line) and that of the Turkish one (black line) in the chemical shift region of amino acids. In the figure, all the identified metabolites are numbered and the most evident spectral differences are highlighted by means of rectangular shapes. In detail, in Turkish lemon there is a greater amount of both saturated (1) and unsaturated (6) fatty acids, lactic acid (8), arginine (10), and *γ*-aminobutyric acid or GABA (13). In contrast, asparagine (21) and malic acid signals (20), even if their peaks are cut in the figure, are more intense in PGI Interdonato lemon of Messina.

In lemon juice the major contribution of unsaturated fatty acids comes from oleic, linoleic, and linolenic acids whereas that of saturated fatty acids comes from palmitic and stearic acids [35]. Organic acids such as citric, isocitric, and lactic acids mainly contribute to determine the lemon acidity that plays the major role in the criteria assessing the commercial acceptability of the fruit. Citric acid (peaks in Figure 4 at 2.85 and 3.00 ppm cut because of their extreme intensity) content in lemon juice is about 5% to 6% [35].

**Figure 5:**
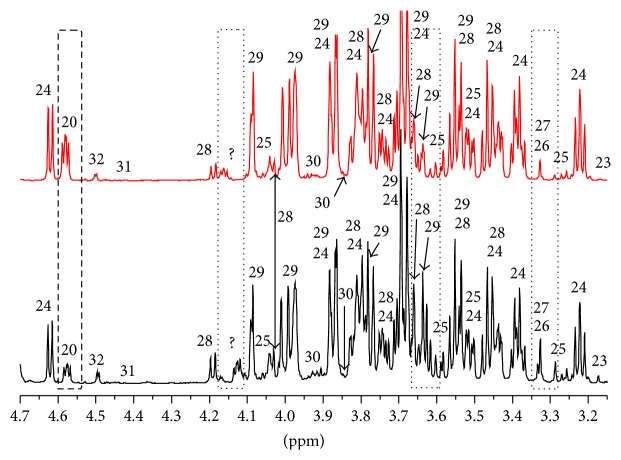
Comparison between the one-dimensional proton spectra of the lemon juice from PGI Interdonato lemon of Messina (red line) and Interdonato Turkish lemon (black line) in the region of the sugars. 20: malic acid, 23: choline, 24: glucose, 25: myoinositol, 26: scyllo-inositol, 27: methanol, 28: sucrose, 29: fructose, 30: serine, 31: trigonelline, and 32: ascorbic acid.

For what concerns the sugars, namely, sucrose, fructose, and glucose, that represent the major component of carbohydrates in citrus fruits and hold the key to sweetness of the juice, the spectral comparison is reported in Figure 5. Fructose and *β*-glucose have a higher concentration in PGI Interdonato lemon of Messina whereas sucrose content is essentially identical. Another metabolite that is very important for nutritional consideration and that displays the same concentration in the two hybrids is vitamin C. The question mark at about 4.15 ppm in Figure 5 represents a metabolite that displays different chemical shift and that we were not able to assign with certainty but should belong to some malonic compounds.

Some minor but important metabolites that we were able to assign are myoinositol (peaks at about 3.28, 3.53, 3.62, and 4.06 ppm), scyllo-inositol (peak at about 3.34 ppm), and stachydrine (peak at about 3.11 ppm). Inositols are present in many vegetable species as minor components and have a positive physiologically activity in human [36]. Many important studies have demonstrated the importance of inositols in the treatment of several diseases such as the polycystic ovary syndrome [37]. Furthermore, myoinositol content and myoinositol/fructose ratio have been found to provide information on the quality and genuineness of orange juice [38]. Stachydrine is an osmoprotectant or osmoprotective compound, which helps organisms to survive extreme osmotic stress [39]. Our evaluation of stachydrine content (about 0.6 mM) (see Table 2) in both Interdonato lemon juices agrees with that of a recent work on the effect of stachydrine on endothelial cell senescence under high glucose stimulation [39]. Finally, the methanol peak at about 3.36 ppm is well evident in the spectrum of Interdonato Turkish lemon but it is not so intense in that of the PGI Interdonato lemon of Messina. Even if we are dealing with low concentrations we want to stress that an excess of methanol is not well tolerated by the human body since it interferes with liver metabolism where it is oxidized.

**Figure 6:**
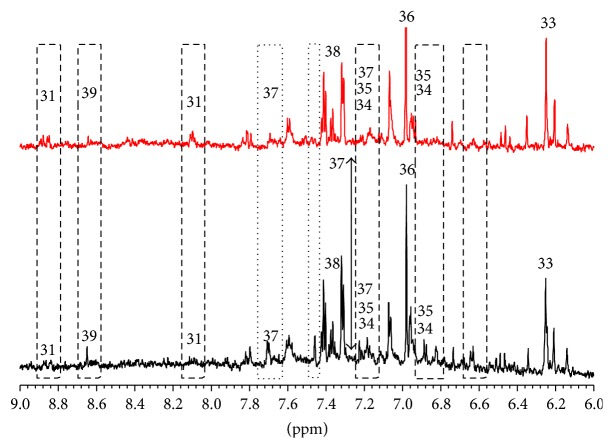
Comparison between the one-dimensional proton spectra of the lemon juice from PGI Interdonato lemon of Messina (red line) and Interdonato Turkish lemon (black line) in the region of phenolic compounds. 31: trigonelline, 33: nucleosides, 34: tyrosine, 35: tyramine, 36: gallic acid, 37: tryptophan, 38: phenylalanine, and 39: adenosine monophosphate (AMP).

Also in the phenolic region (Figure 6) we were able to identify and quantify a good number of metabolites. Here we observe signals coming from nucleosides compounds (33), trigonelline (31), tryptophan (37), tyrosine (34), phenylalanine (38), gallic acid (38), and so forth. Note that phenolic compounds possess antitumoral and health properties [40, 41]. In particular, we want to stress the relatively high concentration, for both hybrids, of gallic acid which is a hydroxybenzoic acid present in food of plant origin and exhibit antioxidative properties [42]. Gallic acid is one example of metabolite identified by means of HSQC experiments.

In Table 2, we report the molar concentration, together with the standard deviation, of those metabolites whose *p* value is below 0.005 and so that can be taken into account for sample differentiation. In particular, the metabolic differences can be considered due to the different geographical origin of the two hybrids of Interdonato lemon.

### 3.3. PDO Extra Virgin Olive Oil of Sicily

Olive oil is the principal source of fat in the Mediterranean diet. Olive oil contains a large proportion of monounsaturated fat, is relatively low in saturated fat, and is another source of the antioxidant vitamin E [43]. These characteristics make olive oil preferable to animal fats just from the standpoint of health [44, 45]. In fact, diets high in monounsaturated fat seem to reduce the risk of atherogenesis and coronary heart diseases, because they increase the concentration of high-density lipoproteins (HDLs) without increasing that of the low-density lipoproteins (LDLs) [46].

Several cultivars of Sicilian eVOOs have been certified with the Protected Designation of Origin (PDO) certificate by the European Commission such as Valdemone, Valle del Belice, Valli Trapanesi, and Val di Mazara. We aim to study their peculiar characteristics in terms of fatty acids concentration and of minor compounds such as terpenes and aldehydes [47].

We have studied the fat composition of several eVOOs produced in Sicily by means of proton HR-MAS NMR, by means of two different but almost equivalent NMR methodologies (based on peak integration) described in Section 2.4. We obtained essentially the same results, for both methods (NMR1 and NMR2) that are reported in Table 3 and compared with the corresponding values obtained by means of Gas Chromatography on some of them [48]. The results are very promising and confirm that ^1^H NMR spectroscopy can be considered a very useful tool for assessing virgin olive oil quality and genuineness [49].

**Figure 7:**
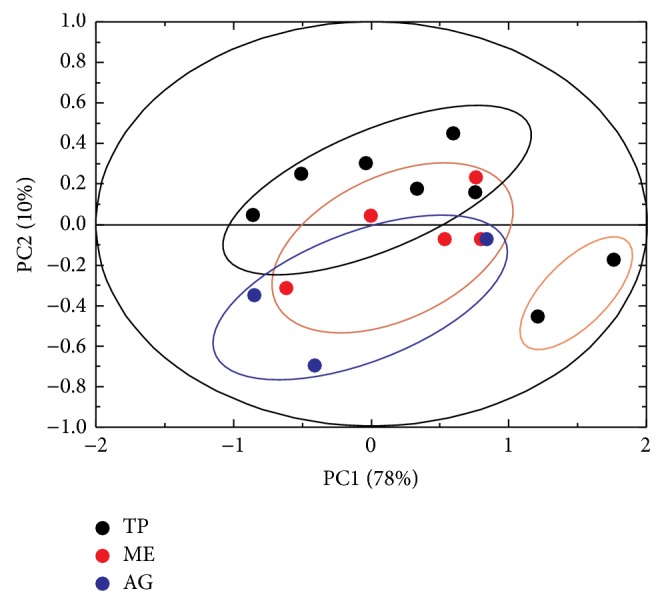
Score plot of the PCA analysis performed on Sicilian eVOOs samples. The results from different Sicilian regions are superimposed to each other; however the orange ellipse on the bottom right side refers to two Spanish cultivars grown in the province of Trapani: Arbosana and Arbequina.

Furthermore, we investigate the possibility to discriminate between samples coming from different Sicilian provinces. To this purpose, we executed the PCA analysis on the processed spectra and the corresponding score plot is reported in Figure 7. Even though there is no clear distinction between different Sicilian regions, being the results superimposed to each other, the two samples grown in the province of Trapani, highlighted with an orange ellipse, belong to two Spanish cultivars: Arbosana and Arbequina.

**Figure 8:**
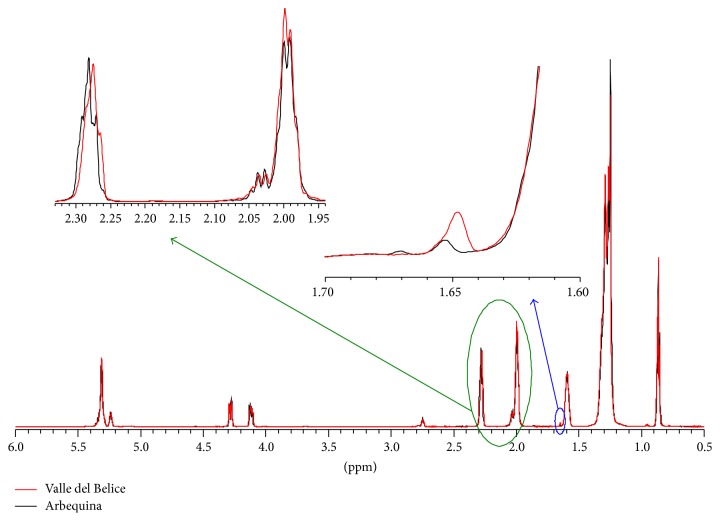
The comparison between the HR-MAS NMR spectra of Arbequina and Valle del Belice cultivars. The expansion on the left side (green arrow) shows that the peak at about 2.83 ppm has the same intensity for the two cultivars, whereas those at about 2.02 ppm and 2.83 ppm are more intense for the Valle del Belice sample rather than for Arbequina.

These two samples show a minor concentration of oleic acid and a major concentration of saturated fatty acids with respect to the original Sicilian cultivars. It is noteworthy that oleic acid is considered to be antithrombotic compared with saturated fatty acids [45]. Furthermore, Arbosana and Arbequina eVOOs possess a minor amount of squalene as pointed out by Figure 8 where the comparison between the spectra of Arbequina and Valle del Belice cultivars is reported. Indeed in Figure 8 the expansion on the left side (green arrow) shows that even though the peak at about 2.83 ppm (representing the total amount of fatty acids) (see Methods Section 2.4) has the same intensity for the two cultivars, that at about 2.02 ppm is more intense for the Valle del Belice sample rather than for Arbequina. Moreover, the expansion on the right side (blue arrow) shows that the peak at about 2.83 ppm, corresponding to squalene, is more intense for Valle del Belice rather than for Arbequina. Squalene is a hydrocarbon and a triterpene involved in the synthesis of all plant and animal sterols. It is known that squalene assumption for olive oil consumption reduces the risk of cancer [50].

### 3.4. Red Garlic of Nubia

Garlic (*Allium sativum L.*) and garlic supplements are consumed in many cultures for their healthy effects. Since the ancient times, garlic was consumed as a remedy for different alimentary disorders and infections [51]. In fact, in literature, there are many studies that investigate garlic preparations and their properties [52].

**Figure 9:**
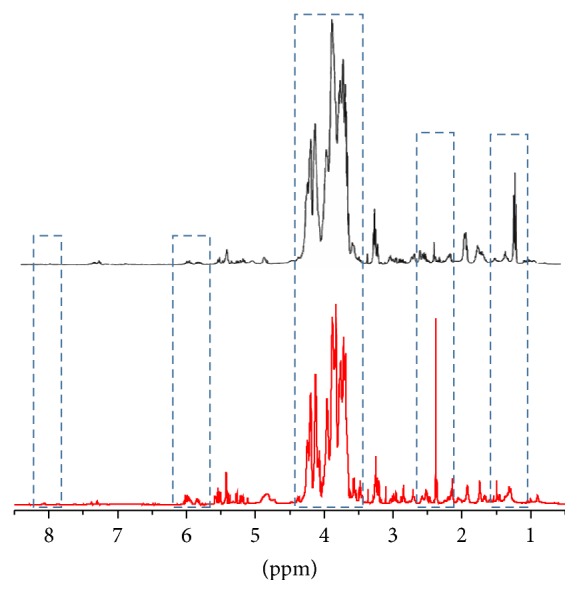
The comparison between the HR-MAS NMR spectra, of two different experiments on two red garlic samples: the red garlic of Nubia (red line, measured by us with a 700 MHz spectrometer) and the red garlic of Sulmona (black line, measured by Ritota et al. [16] with a 400 MHz spectrometer). The highlighted regions represent the most important similarities and differences.

The most important garlic compounds that have beneficial effects on human health are the organosulphur ones [52]. The peculiar garlic fragrance arises from allicin and other oil-soluble sulfur components. However, once garlic is cut or crushed, compounds in the intact garlic are converted into hundreds of organosulfur compounds in a short period of time. For example, alliin is converted to allicin by alliinase. Allicin is an effective antimicrobial agent that can be found in limited amounts only in freshly crushed garlic [53]. Another important allylic compound is S-Allyl Cysteine (SAC) that has a strong antifungal action and that seems to be highly present just in the red garlic of Nubia (see the region at about 6 ppm of NMR spectra in Figure 9).

We have determined the molar concentration of the main metabolites of the red garlic of Nubia by studying the one-dimensional proton spectrum obtained by means of HR-MAS NMR. The peak assignment was particularly difficult in the carbohydrates and allylic regions of the NMR spectra for the superposition of signals belonging to many similar chemical species. Our results confirm and extend those obtained by means of the same technique by Ritota et al. [16] on white and red Italian garlic. In Figure 9 we report the comparison between the HR-MAS NMR proton spectra of two different Italian red garlics: the red garlic of Nubia (measured by us with a 700 MHz spectrometer) and the red garlic of Sulmona (measured by Ritota et al. [16] with a 400 MHz spectrometer).

In the figure, we have highlighted five different chemical shift regions that are relevant for the comparison. The region centered at about 4 ppm belongs to carbohydrates and is similar for both spectra. The regions centered at about 6 and 8 ppm have instead a different intensity (higher for the Nubia sample) and correspond to allylic compounds and riboflavin, respectively. The major differences between the spectra of the two red garlics are showed in the first two regions. In particular, it is noteworthy that only in the spectrum of the red garlic of Sulmona (black line in Figure 9) there is a sharp triplet at about 1.23 ppm that the authors [16], together with a signal at about 3.95 ppm, assign to an unknown compound. We believe that these signals belong to diethylthiophosphate, that is, an organophosphorus compound widely used as pesticide because of easy degradation in the environment. On the other side, the peak at about 2.4 ppm, belonging to pyruvate, is much more intense in red garlic of Nubia with respect to the other red garlic considered. In the following table (Table 4), we report the molar concentration of the identified metabolites that were quantified by means of the above described procedures. We list only the metabolites whose concentration has a standard deviation less than 15%.

## 4. Conclusions

In this work, we have presented our studies, by means of the powerful NMR technique known as HR-MAS, on the characterization of some food products typical of the Mediterranean diet. In particular, we have analyzed the metabolic profile of the PGI cherry tomato of Pachino and we were able to identify few metabolites that can be considered for sample authentication. For example, in this protected foodstuff, we have found a higher concentration of GABA, sugars (fructose and glucose), glutamic compounds (glutamate and glutamine), and phenolic compounds (trigonelline, tyrosine, and tryptophan) with respect to non-Pachino cherry tomatoes. Furthermore, we have characterized the metabolic profile of juice from the PGI Interdonato lemon of Messina and compared it with that of the juice from the same hybrid cultivated in Turkey. We find for both hybrids high levels of sugars (sucrose, fructose, and glucose), citric acid, vitamin C, gallic acid, and inositols (mioinositol and scyllo-inositol).

The major source of fat in the Mediterranean diet comes from the consumption of eVOOs. For this reason, we have studied the fatty composition of several Sicilian cultivars (including few PDO samples) by means of two different methods both based on peaks integration. The results are consistent with those obtained by means of Gas Chromatography and confirm the power of NMR technique for quick quantitative chemical analysis. Moreover, we were able to discriminate between cultivars grown in the same province (Trapani) but coming from different nations (Italy and Spain), just for the different amount of oleic and unsaturated fatty acids and also for squalene content.

Finally, we have characterized the metabolic profile of the PAT red garlic of Nubia, quantifying the principal metabolites. In addition, we have compared its proton HR-MAS NMR spectrum with that of another Italian red garlic and have revealed that both garlics possess the same amount of carbohydrates. However, the red garlic of Nubia has a bigger amount of riboflavin, pyruvate, and allylic compounds. On the other side, only the red garlic of Sulmona shows a peak that could belong to diethylthiophosphate, a widely used pesticide.

In conclusion, the overall results allow appreciating the enormous potential of the used technique that is able to reveal and quantify a number of metabolites (characteristic of the particular food product condition), even on few amounts of samples and without any chemical treatment. The NMR technique is a rapid (few minutes of signal acquisition), nondestructive (no need of sample treatment), and reliable methodology to be used in an official method eventually in conjunction with other traditional analytical techniques such as GC. We want to stress that the consequence of insisting on NMR spectroscopy for food products characterization leads to the reduction of chemical consumption and waste production, which is important from both the economic and environmental points of view. All these characteristics also make the methodology very attractive for industry.

## Figures and Tables

**Table 1:** Comparison between the statistically significant average metabolite molar concentrations of Pachino cherry tomatoes and of non-Pachino ones. Besides, we signed in bold the metabolites with the higher concentration.

Metabolite	Pachino cherry tomatoes (mM)	Non-Pachino cherry tomatoes (mM)
GABA	**0.89**	0.34
Alanine	0.26	**0.54**
Aspartic acid	**0.81**	0.50
Fructose	**19.4**	14.6
Glucose	**13.4**	10.8
Glutamic acid	**1.26**	0.78
Glutamine	**1.59**	1.04
Guanosine	0.033	**0.098**
Methanol	0.61	**1.35**
Trigonelline	**0.051**	0.023
Tryptophan	**0.068**	0.032
Tyrosine	**0.058**	0.022

**Table 2:** Average metabolites molar concentration of PGI Interdonato lemon of Messina and Interdonato lemon of Turkey juices.

Metabolite	PGI Interdonato lemon of Messina (mM)	Interdonato lemon of Turkey (mM)
Alanine	0.95 ± 0.01	1.49 ± 0.02
AMP	0.016 ± 0.001	0.039 ± 0.002
Arginine	1.1 ± 0.1	1.61 ± 0.02
Asparagine	16.3 ± 1.0	10.8 ± 0.8
Choline	0.032 ± 0.004	0.102 ± 0.014
Fructose	70.4 ± 1.3	38.1 ± 0.6
GABA	1.05 ± 0.05	2.4 ± 0.1
Glucose	65.4 ± 2.0	35.7 ± 1.1
Isoleucine	0.097 ± 0.007	0.287 ± 0.009
Lactic acid	0.11 ± 0.01	0.61 ± 0.02
Leucine	0.177 ± 0.012	0.467 ± 0.028
Malic acid	16.6 ± 1.3	6.9 ± 0.2
Methanol	0.19 ± 0.01	1.08 ± 0.11
Myoinositol	4.8 ± 0.2	3.2 ± 0.3
Phenylalanine	0.17 ± 0.01	0.28 ± 0.01
Proline	0.58 ± 0.02	1.79 ± 0.04
Scyllo-inositol	0.67 ± 0.01	0.96 ± 0.02
Stachydrine	0.59 ± 0.02	0.69 ± 0.025
Threonine	0.27 ± 0.02	0.74 ± 0.02
Tryptophan	0.057 ± 0.006	0.13 ± 0.01
Tyramine	0.019 ± 0.001	0.044 ± 0.003
Tyrosine	0.013 ± 0.001	0.044 ± 0.004
Valine	0.29 ± 0.02	0.55 ± 0.02

**Table 3:** Fatty acids percentage in Sicilian eVOOs determined by the two considered methods compared with that obtained by GC on some of them. See text for labels explanation.

Cultivar	Linolenic acid (E)			Linoleic acid (A)			Oleic acid (C)			Saturated acids		
	NMR1	NMR2	GC	NMR1	NMR2	GC	NMR1	NMR2	GC	NMR1	NMR2	GC
Arbequina (TP)	0.61	0.59	0.64	10.07	10.04	10.39	70.20	68.80	68.42	18.50	20.20	19.45
Arbosana (TP)	0.55	0.55	0.51	6.50	6.47	5.95	74.34	72.95	72.42	19.50	19.50	19.30
Dop Valle del Belice (TP)	0.91	0.93	∖	8.65	8.59	∖	76.45	75.65	∖	14.66	13.88	∖
FSI17 (TP)	1.15	1.14	1.08	8.28	8.24	7.92	75.77	75.13	72.36	15.32	14.35	16.02
Valli Trapanesi (TP)	0.57	0.56	∖	8.69	8.68	∖	78.09	77.69	∖	12.83	12.51	∖
Nocellara del Belice (TP)	0.73	0.72	∖	9.53	9.34	∖	77.73	75.94	∖	14.01	13.27	∖
Nocellara del Belice 2 (TP)	0.51	0.50	∖	8.26	8.16	∖	78.65	77.45	∖	13.79	13.38	∖
Nocellara del Belice 3 (TP)	0.56	0.56	0.55	8.94	9.44	9.80	77.20	77.10	76.70	13.30	12.90	12.95
Santagatese (ME)	0.76	0.73	∖	9.77	9.45	∖	78.61	75.84	∖	14.17	13.24	∖
Ogliarola Messinese (ME)	0.67	0.65	∖	7.04	6.89	∖	82.09	80.06	∖	12.41	11.75	∖
Acquedolci Santagatese (ME)	1.08	1.01	∖	15.51	14.83	∖	71.54	68.16	∖	16.46	14.97	∖
Dop Valdemone (ME)	0.59	0.58	∖	9.17	9.10	∖	79.23	78.43	∖	11.71	11.31	∖
Dop Valdemone 2 (ME)	0.94	0.93	∖	8.49	8.34	∖	79.65	77.99	∖	12.69	11.80	∖
Cerasuola (AG)	0.59	0.61	0.58	10.25	10.15	10.63	77.00	76.90	76.16	12.16	12.34	12.62
Biancolilla 1 (AG)	0.86	0.83	0.78	9.22	8.99	9.64	75.72	73.62	71.56	16.71	15.73	16.47
Biancolilla 2 (AG)	0.82	0.80	∖	10.01	9.80	∖	76.00	74.10	∖	15.40	14.50	∖

**Table 4:** Average metabolites molar concentration of red garlic of Nubia.

Metabolite	mM
2-Phenylpropionate	0.9
4-Aminobutyrate	1.36
Alanine	0.65
Arginine	12.6
Ascorbate	1.2
Asparagine	10.3
Aspartate	0.88
Caprate	1.6
Choline	1.1
Citrate	3.03
Cystine	0.33
Ethanol	0.97
Formate	0.12
Fructose	15.4
Fumarate	0.049
Glucose	0.98
Glutamate	5.1
Glutamine	6.4
Glycine	4.7
Histidine	0.14
Isoleucine	0.37
Leucine	0.45
Lysine	1.02
Malate	1.45
Malonate	0.16
Maltose	0.4
Methanol	2.16
Methionine	0.33
Nicotinate	0.004
O-Phosphocholine	0.82
Pantothenate	0.19
Phenylalanine	0.11
Proline	1.5
Pyruvate	10.4
Riboflavin	0.87
Serine	30.1
Sucrose	21.0
Threonine	1.3
Thymine	0.25
Trigonelline	0.3
Tryptophan	0.06
UDP-glucose	0.073
Valine	0.63
